# Breast and Cervical Cancer Screening among US and non US Born African American Muslim Women in New York City

**DOI:** 10.3934/publichealth.2017.1.78

**Published:** 2017-02-21

**Authors:** Karent Zorogastua, Pathu Sriphanlop, Alyssa Reich, Sarah Aly, Aminata Cisse, Lina Jandorf

**Affiliations:** 1 Department of Oncological Sciences, Icahn School of Medicine at Mount Sinai, New York, NY, United States; 2 Department of Genetics and Genomic Sciences, Icahn School of Medicine at Mount Sinai, New York, NY, United States

**Keywords:** breast cancer, cervical cancer, cancer screenings, Muslim women, African women

## Abstract

**Purpose:**

Health disparities related to breast and cervical cancer among African American and African-born Muslim women in the United States have been identified in previous literature. Our study aimed at exploring the breast and cervical screening rates and factors that influence this population's disposition to adhere to cancer screening exams.

**Methods:**

Mixed methods were used to collect data with African American and African-born Muslim women in New York City. Data were collected from a total of 140 women; among them, 40 participated in four focus groups.

**Findings:**

Focus groups revealed nine themes: healthcare practices; lack of knowledge/misconceptions; negative perceptions and fear; time; modesty; role of religion; role of men; role of community; stigma and shame. Among 130 women who reported their cancer screening status, 72.3% of those age 21 and over were adherent to cervical cancer screening; 20.0% never had a Pap test. Among women age 40 and over, 80.2% reported adherence to recommended mammogram; 12.8% never had one. Among women under age 40, 52.2% had their last clinical breast exam (CBE) less than three years ago. Among women age 40 and over, 75.0% were adherent to yearly CBE.

**Conclusions:**

While rates of screenings were above the national average and higher than expected, specific barriers and facilitators related to religious and health beliefs and attitudes that influence the decision to adhere to screening were revealed. These factors should be further explored and addressed to inform future research and strategies for promoting regular breast and cervical cancer screenings.

## 1. Introduction

Breast cancer is the most commonly diagnosed cancer among women in the United States (US), regardless of race or ethnicity [1]. Early detection of breast cancer through clinical breast exams (CBE) and mammography is key to reducing morbidity and mortality. Increased mammography screening has contributed to the nearly 20% decline in breast cancer mortality in the past 20 years [2]–[5]. Nonetheless, higher rates of breast cancer incidence continue to persist for minority groups largely due to lower rates of breast cancer screening and delayed follow-up of abnormal screening results [6]–[8].

Similarly, cervical cancer continues to be ranked as a common cancer among US women at 14th in frequency [9]. Much like breast cancer, cervical cancer incidence and deaths in the US have decreased significantly over the past 40 years as a result of increased screening in the form of regular Papanicolaou (Pap) tests [10]. However, the rates of new cases of cervical cancer as well as the death rates are higher among certain race/ethnicities, particularly among Black women [11]. The majority of invasive cervical cancer cases occur among women who do not adhere to timely cervical cancer screenings [12],[13]. It has been estimated that as many as 80% of cervical cancer deaths could be prevented by regular screening coupled with adequate follow-up and treatment [14].

Both US-born and immigrant Muslim women in the US represent a unique and fast growing population whose health behaviors and determinants have rarely been studied [15]–[20]. Among the few studies that have measured mammography screening rates among Muslims in the US, nearly all have reported rates of biennial mammography lower than the national averages of 67% for women over 40 years and the Healthy People 2020 target of 81% [21],[22]. In a sample of 207 South Asian and Middle Eastern Muslim women from the Chicago area, Hasnain and colleagues [15] found that only 52% of women reported mammography screening in the past two years [23]. Another study of 240 diverse American Muslim women in Chicago reported 58-63% adherence to mammography guidelines [24],[25]. Lower mammography rates have also been found among Muslim women in Southern California where only 54% reported having a mammogram in the past two years [20] and in Detroit where the rate for Muslim women was only 58% [26].

Cervical cancer screening rates among religious minorities in the US, including Muslim women, are largely unknown. Among the few studies that have surveyed cervical cancer screening rates among Muslim women, low rates have been found. The national average of women who had a Pap test within the past three years is 73%; the Healthy People 2020 target is 93% [27],[28]. Among a sample of 50 Arab Muslim women in Pennsylvania, it was found that only half had received Pap testing within the previous three years [29]. Among 247 Muslim women from the Chicago area, 84% had received a pap in the last three years [30]. While Padela and colleagues [30] demonstrate a Pap test rate higher than that of the national average, it demonstrates an average lower than that being targeted by Healthy People 2020.

It has been suggested that Muslim women's healthcare behaviors, including cancer screening practices, are significantly influenced by religious beliefs and experiences [15]–[19],[31]–[36]. Yet, the relationships between religious beliefs, related factors, and screening practices are not well elucidated [30]. In addition, multiple factors that contribute to barriers faced by minority women in the US to healthcare including language barriers, lack of medical insurance, geographical barriers, limited knowledge, lack of education, and lack of access to healthcare services [37] have not been well studied among Muslim women in the US.

Aims and Objectives

This study aimed to answer the questions: What are the breast and cervical cancer screening rates among African American and African-born Muslim women? What are the barriers and facilitators to breast and cervical cancer screening among this population? The number of Muslims in the US is estimated to be between five and seven million, with approximately 700,000 in New York City (NYC), one of the highest concentrations of Muslim in the US [38]. Among US-born Muslims in NYC, 20–25% are African American [38]. Given the health disparities related to breast and cervical cancer screening, it is essential to understand the disparities and factors that influence the growing African American and African-born Muslim population's disposition to engage and adhere to cancer screening exams.

## 2. Materials and Methods

Mixed methods were used due to the exploratory nature of this study. Qualitative and quantitative data were collected through focus groups and individual questionnaires with self-identified African American and African-born Muslim women in NYC. Focus groups and questionnaires aimed at eliciting screening knowledge and behaviors as well as barriers to breast and/or cervical cancer screening, particularly those related to their cultural and religious beliefs.

Institutional Review Board approval was obtained from the representative academic institution, the Icahn School of Medicine at Mount Sinai. All participants who partook in the focus group signed consent forms. Since no identifying information was being obtained, the Institutional Review Board approved that participants who solely completed the demographic surveys were only required to provide verbal consent.

In order to obtain screening and health behavior information strictly from our population of interest, screening methods were carried out by recruiters asking potential participants if they self-reported as female, Muslim, and African American or African-born.

Sample and Data Collection

Through convenience sampling, 140 self-identified African American and African-born Muslim women over the age of 18 participated in the study; 40 participated in the focus groups and survey, while 100 participated in the survey alone. To address language barriers, all materials were translated and back translated into Arabic and French, the leading secondary languages among African Muslims in NYC. Although participants had the option of participating in French, Arabic, or English language focus groups, all were conducted in English.

Recruitment sites included NYC faith-based institutions, community agencies, workplaces, and neighborhood commercial markets and stores. Participants were recruited through word-of-mouth, flyers, and in-person at community centers. Focus group participants were given a $20 gift card and those who participated in the survey alone were given a $5 (roundtrip) NYC Metrocard for their participation. Focus groups lasted about two hours and were audio recorded. Focus groups were conducted until saturation of themes was reached.

The primary outcomes included self-reported adherence to mammogram, CBE, and/or Pap test. Focus groups elicited qualitative responses regarding knowledge of breast and cervical cancer, prevention and healthcare practices, attitudes and beliefs related to cancer screenings, and cultural, logistical, and systemic barriers and facilitators to obtaining healthcare. The quantitative surveys collected demographic characteristics such as education level, income, marital status, years in the US, language preference(s), and country of origin. The American Cancer Society recommendations at the time were used to define adherence: CBE screening every three years for women in their 20s and 30s and every year starting at age 40; mammography every year beginning at age 40; and Pap test every three years starting at age 21 [39]–[41].

## 3. Results

### 3.1. Focus Groups

Four focus groups were conducted with 40 Muslim women. The qualitative data were subjected to thematic analysis. Two research assistants independently read all of the transcripts to develop a thematic schema. The research team met regularly to discuss interpretations, refine themes, and developed a final outline.

The analysis of the qualitative data was guided by the Social Ecological Model (SEM) and involved identifying patterns in the transcripts, coding the data, and identifying themes [42]. To facilitate a multi-level approach for future intervention, qualitative data were categorized according to the hierarchical domains of the SEM. There are five domains of the SEM: Individual; Interpersonal; Community; Organizational; and Policy/Enabling Environment.

**Figure 1.**
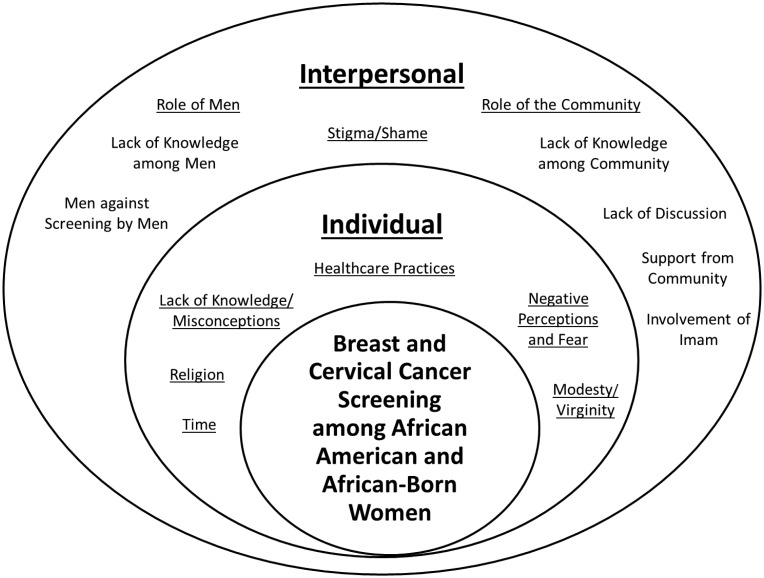
Interrelation of Domains and Themes.

Nine themes pertaining to breast and cervical cancer screening among African American and African-born Muslim women were identified and divided across two domains of the SEM, Individual Factors and Interpersonal Factors (Table 1). The interrelations of themes and domains are illustrated in Figure 1.

**Table 1.** Qualitative Themes.

Domains	Themes
**Individual Factors**	**Healthcare Practices**
	i) Seeks Medical Attention only when Necessary
	ii) Alternative Medicine
	iii) Medical Mistrust
	**Lack of Knowledge/Misconceptions**
	Breast Cancer
	i) Contagious
	ii) Caused by Mammogram
	Breast Cancer Screenings
	i) Age
	ii) Not Every Year
	iii) Only when using Contraceptives
	Cervical Cancer
	Cervical Cancer Screenings
	i) Starts at Menarche
	ii) Only Done when Pregnant
	**Negative Perceptions and Fear**
	i) Breast Cancer as Deadly
	ii) Breast Cancer is a Serious Diagnosis
	**Time**
	**Modesty**
	**Role of Religion**
	i) Refusal due to God's Plans
	ii) Religion not a Hindrance
**Interpersonal Factors**	**Role of Men**
	i) Lack of Knowledge/Misconceptions Among Men
	ii) Against Screening Exams by Male Providers
	**Role of the Community**
	i) Lack of Knowledge/Misconceptions
	ii) Lack of Discussion
	iii) Support from Community
	iv) Involvement of the Imam
	**Stigma and Shame**
	i) Stigma from the Community
	ii) Shame among the Afflicted
	iii) Shame among Men

#### 3.1.1. Individual Factors

Within the Individual domain, healthcare practices arose as a predominant theme and barrier to adhering to breast and cervical cancer screening. While women expressed that they had access to care, some women responded that they did not have a regular healthcare provider and only sought care when necessary. Women also explained that some practiced alternative medicine, engaging in the use of natural medicines and healers.

“I'm one of those persons. I don't go unless I'm sick.”

Medical mistrust also emerged as a subtheme. Women expressed that they particularly had concerns with male providers.

“Some (Muslim women) don't like men doctors.”

Lack of knowledge or misconceptions about breast and cervical cancer and screening also emerged as a barrier to screening adherence. In relation to breast cancer, some women believed it is contagious.

“… Some people think you can contract it because your sister has it or your friend has it.”

Others believed the radiation from mammograms could cause cancer.

“… (Muslim women) don't want to do it. They say the radiation makes you get cancer.”

Women also revealed a lack of knowledge or misunderstanding about breast cancer screening guidelines. Some women were confused about what age to begin screening. A subset of women also believed that regular breast cancer screening is only necessary when women take oral contraceptives.

Women also expressed a lack of knowledge or misconceptions about cervical cancer and screenings. Women explained that they did not have enough information about cervical cancer and that in terms of screening, there were mistakenly different points at which a woman should start screening- at menarche, when sexual activity begins, or when pregnant.

Negative perceptions and fear also seemed to hinder women from getting screened. Women stated that they viewed breast cancer as deadly.

“I know it's a bad sickness and it kills a lot of people…, it killed my aunt.”

Time also arose as a barrier to adhering to cancer screenings. Women reported that the main issues with time were due to work and family.

Concerns over modesty and religion in relation to cancer screening, particularly for cervical cancer, were expressed. Women believed that a Pap test was not to be performed until women are married.

“In my culture, a woman should be pure. I suggest it be done after she loses her virginity.”

Similarly, the role that religion played in the decision to screen was a predominant theme. Women explained that Muslims refuse to be screened because the chances of getting cancer are in the hands of Allah.

“…I think (the) attitude some people in our faith … (have is), ‘Oh, this is God's plan.’”

However, some women in the group countered this notion explaining that their religious beliefs were not a hindrance to screening.

“We know that Allah is the master planner…but we know also that he allows men to create healing.”

#### 3.1.2. Interpersonal Factors

The role of men was a leading theme that seemed to be a factor in women's decision to engage in breast and cervical cancer screening. Women expressed that men had a lack of knowledge about screening that needed to be addressed.

“I think there's a certain type of man that is engaged in the healthcare of his family…, but most…I don't think they know.”

They also expressed that some Muslim men were against screenings, particularly by male providers.

The role of the African Muslim community in the decision to screen was also a major theme. However, women explained that there was a lack of knowledge as well as discussion about breast and cervical cancer in the community, particularly among youths. Nonetheless, women explained that there is support from the community especially if a member is diagnosed with cancer.

“…if you're sick … go to the Mosque, go to the community center so they can help you.”

They also expressed how support from the Imam, their religious leader, is essential and that he should be engaged in the discussion about screenings.

“…everything (the Imam) says, you believe….get them involved.”

Despite the sense of support expressed, some women explained that stigma and shame about cancer still exist in the community, particularly among men. The community's reaction to a diagnosis depended on the type of cancer.

“It's shame to say that I got cancer.”

“What scares me is that men are walking around with cancer and they're in denial.”

### 3.2. Surveys

There were no differences in demographic characteristics or screening statuses between women who participated in the focus groups (n = 40) and those who participated in the surveys only (n = 100). Participants represented over 18 countries of birth. The most common countries of origin represented included the Ivory Coast (20.7%), Senegal (15.7%), the US (10.0%), Mali (10.0%), and Morocco (9.3%). The average age was 42.8 years, 51.8% were married or living with a partner, 24.5% were unemployed, 42.9% had less than a high school degree, and 33.6% had an annual household income of less than $10,000. The majority of participants reported they were very religious (67.1%) and almost all women (96.4%) reported attending religious services at least once a week. The majority of participants (90.0%) were not born in the US. Among the foreign-born, the average amount of time in the US was 14.4 years. There were no differences in demographic characteristics or screening statuses of participants who were born in the US compared to those born outside the US. The demographic characteristics are presented in Table 2.

Participants were also asked to report their breast and cervical cancer screening status and, if screened, report the date of their last CBE, mammogram, and Pap test (see Table 3).

**Table 2.** Demographic Factors.

	N = 140
**Age**	*n = 139*
Minimum	18
Maximum	70
Mean (SD)	42.8 (9.8)
**Location of Residence**	*n = 140*
Manhattan	56 (40.0%)
Brooklyn	13 (9.3%)
Staten Island	1 (0.7%)
Bronx	66 (47.1%)
Queens	2 (1.4%)
Outside of NYC	2 (1.4%)
**Marital Status**	*n = 139*
Married or Living with Partner	72 (51.8%)
Single, Divorced, or Widowed	67 (48.2%)
**Currently Employed**	*n = 139*
No	34 (24.5%)
Yes, Part-Time	61 (43.9%)
Yes, Full-Time	44 (31.6%)
**Education**	*n = 136*
Less than High School	60 (42.9%)
High School Degree or GED	38 (27.1%)
Some College or More	35 (25.0%)
**Total Income**	*n = 130*
Less than $10,000	47 (33.6%)
$10,00 to $24,999	41 (29.3%)
More than $25,000	42 (30.0%)
**Country of Origin**	*n = 140*
Gambia	5 (3.6%)
Guinea	14 (10.0%)
Ivory Coast	29 (20.7%)
Mali	14 (10.0%)
Morocco	13 (9.3%)
Senegal	22 (15.7%)
United States	14 (10.0%)
Other African countries	39 (27.9%)
**How long have you been in the US? (Years)**	*n = 118*
Mean (SD)	14.4 (7.7)
**English Proficiency**	*n = 134*
Yes	93 (69.4%)
No	41 (30.6%)
**How religious are you?**	*n = 140*
Not at all or a little religious	6 (4.3%)
Somewhat religious	18 (12.9%)
Pretty or very religious	116 (82.9%)
**How often do you attend religious service in a week? (Days)**	*n = 140*
Minimum	0
Maximum	7
Mean (SD)	2.6 (2.2)
**Health Insurance Status**	*n = 138*
No Insurance	26 (18.8%)
Medicaid	69 (50.0%)
Medicare	8 (5.8%)
Private	19 (13.8%)
Other	8 (5.8%)
Don't Know	8 (5.8%)

**Table 3.** Screening Statuses.

	N = 140
**Pap Test (Women 21 and Over)**	*n = 130*
Adherent	94 (72.3%)
Non-adherent (never had one)	26 (20.0%)
Had one over 3 years ago	10 (7.7%)
**Mammogram (Women 40 and Over)**	*n = 86*
Adherent	69 (80.2%)
Never had one	11 (12.8%)
Last mammogram over a year ago	6 (7.0%)
**CBE (Women Under 40)**	*n = 46*
Adherent	24 (52.2%)
Never had one	21 (45.7%)
Last CBE over 3 years ago	1 (2.2%)
**CBE (Women 40 and Over)**	*n = 88*
Adherent	66 (75.0%)
Never had one	18 (37.5%)
Last CBE over a year ago	4 (4.5%)

Among women under age 40, 52.2% reported having their last CBE less than three years ago. Among women age 40 and over, 75.0% reported being adherent to yearly CBE. Among women age 40 and over, 80.2% reported adherence to yearly mammogram and 12.8% reported never having had one. Among women age 21 and over in our sample who responded, 72.3% were adherent to cervical cancer screening and 20.0% reported never having had a Pap test.

## 4. Discussion

Adherence to breast cancer screening via mammography and cervical cancer screening were both higher among our sample compared to that of the national average. In addition, neither socio-cultural predictors to screening including marital status, country of origin, length of time in the US, English proficiency, level of religiosity, nor socio-economic predictors to screening including employment status, level of education, income, or health insurance status were found. However, focus groups not only revealed notable barriers to screening including lack of knowledge and the cultural issue of modesty but also the different levels of influence that family, friends, and community play among women in the African Muslim community on health behavior.

One possible explanation for the lack of knowledge about breast and cervical cancer and screening guidelines among our sample may lie in that cancer is not widely spoken about or is stigmatized in the Muslim community. In a study conducted with Arabic women in Qatar, less than 3% of women were determined to have a “basic” understanding of breast cancer [43]. Most of the women in Donnelley and colleague's study who knew about breast cancer and how to screen for it heard of it via discussion and word of mouth [44]. Women who attended our focus groups reported there was a lack of discussion in their community, particularly among youth. Being that a majority of the women in our sample were from the same communities, perhaps they realized the benefit of sharing important health information within their circle.

Donnelly and colleagues [44] also found that greater knowledge of breast cancer and screenings was positively correlated with higher screening rates. Those who participated in our study who were both proficient in English and employed had relatively high screening rates, despite their country of origin. Perhaps the two factors, language and employment, may speak to their acculturation and attained knowledge of “Western” medicine's view on cancer and screenings.

The barrier of modesty was also highlighted in many of the women's views regarding Pap tests (i.e., when to have them, who should examine them). This barrier is universal to Muslim women regardless of ethnicity [45]. Particularly, having a male doctor may prove as barrier in healthcare settings, especially when it comes to screening for cervical cancer.

### 4.1. Implications

When conducting further research, program development and implementation with the African Muslim population, inclusion of family and community members, particularly Imams and males, should be a priority. Our research revealed the importance that community members, particularly religious leaders, play in not only carrying out our research but in also determining the actions and behaviors of the women in our sample.

In addition, other areas of research should be explored among this population including the correlation between religiosity level and screening behavior. For example, women from more religious backgrounds may be opposed to screening due to modesty, a theme that commonly arose within the focus groups.

Similarly, the effect of healthcare accommodation, including racial, ethnic, or gender concordance, and provider knowledge of cultural practices on the uptake of cancer screening needs to be explored. More research may lead to improved practices for Muslim women. For example, attention can be given to the gender of the doctor providing a breast exam or Pap test. Additionally, the inclusion of a female chaperone or female family member in an exam room may remove a barrier to screening for some Muslim women.

Finally, the differences in needs dependent on acculturation should also be further explored. For example, older women who are less acculturated may have barriers to screening that include language differences or practices in alternative medicine. However, for younger adults, a lack of knowledge and a lack of education may be more relevant barriers.

### 4.2. Limitations

Our study's conclusions are limited by several factors. Firstly, it is important to note that this is a preliminary study with a sample of convenience which may not be representative of all African American and African-born Muslims in NYC. Thus, we cannot generalize our findings to reflect the views of women in NYC and that of other cities in the US or globally. However, we feel that this is an important contribution and first step to better understand the needs of this growing minority population.

Our sample represented a diverse set of African countries with Muslim communities that speak different languages and dialects. Demographic surveys were translated into Arabic and French, and although participants had the option of participating in the focus groups in French, focus groups were only conducted in English. This may reflect the relative degree of acculturation of the women who participated in the focus groups. Those who were comfortable participating in the focus groups in English may approach their medical care in alignment with more “Western” approaches to medicine. As a result, it is possible that we did not reach African American and African-born Muslim women who had lower screening rates due to lower levels of acculturation, lower level of English proficiency, and who consequently may be at greater risk and experience more barriers to screening.

Winslow and colleagues [46] recommend the use of same-gender and same-culture interviewers for qualitative research in studies. Although focus group facilitators and moderators were female, not all were culturally concordant. Similarly, research assistants and coordinators who conducted the surveys were all female but not all were Muslim, African American or African-born. As a result, women in our study may not have been as open to share their story with someone of a different race, religion, or culture. Although we worked with the religious leaders (i.e., Imams), the fact that our research team was not from within the faith may have also biased our results.

Lastly, we found higher than expected rates of cancer screenings. This may be due to the self-reported nature of the data such that healthcare behavior may have been over- or under-reported. However, we are also aware that we might not have been able to reach those women who are less likely to be screened such as less acculturated women, those who are the most traditional in their beliefs, and those who were not permitted to participate by their male family members.

Despite these limitations, our study has several strengths. First, although a convenience sample of African American and African-born Muslim women from NYC was used in this study, it is important to note that NYC represents a large US city that is a haven to many minority and immigrant communities, similar to that of many other large US cities. Although, different results may be produced when looking at African Muslim communities that live in smaller or less diverse areas, focusing on those within NYC may provide a good representation of those in other major immigrant and minority centers.

Second, this is one of few studies, to our knowledge, that has examined the screening rates and cultural specific barriers and facilitators to engage or not engage in breast and cervical cancer screening among African American and African-born Muslim women in the US, a growing yet vulnerable population. In addition, this study is one of the few, if any, that uses the SEM to not only identify primary influences on breast and cervical screening adherence or nonadherence among this population, but to also inform future research, program development, and clinical care.
